# Teaching Neuro*Images*: Nonfluent variant primary progressive aphasia: A distinctive clinico-anatomical syndrome

**DOI:** 10.1212/WNL.0000000000003408

**Published:** 2016-12-06

**Authors:** Charles R. Marshall, Christopher J.D. Hardy, Martin N. Rossor, Jason D. Warren

**Affiliations:** From the Dementia Research Centre, University College London, UK.

A 66-year-old woman presented with 4 years of progressive speech difficulty. She had nonfluent speech with phonemic errors but intact single-word comprehension and object knowledge. Her grammar was impaired in both speech and writing, and she exhibited orofacial apraxia. A clinico-radiologic (see figure) diagnosis of nonfluent variant primary progressive aphasia was made.

**Figure:**
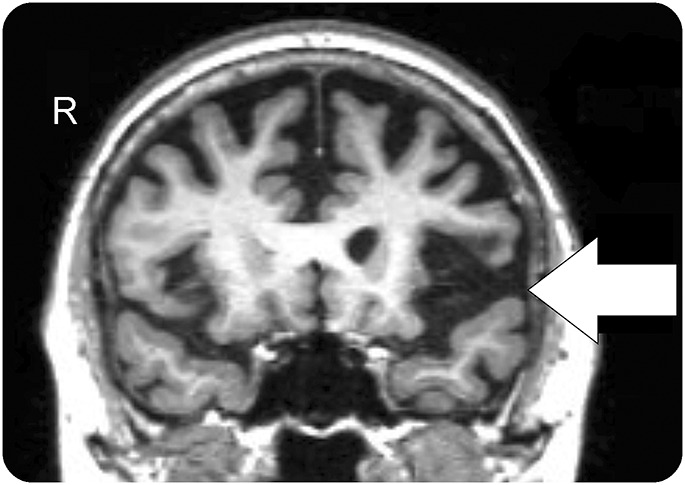
Magnetic resonance image Coronal volumetric T1-weighted MRI showing asymmetric atrophy of left insula and opercular inferior frontal gyrus (arrow), a pattern typical of nonfluent variant primary progressive aphasia.

Nonfluent variant primary progressive aphasia is a neurodegenerative disease within the spectrum of frontotemporal dementia, characterized by the typical language and brain atrophy patterns seen here.^1^ It is most frequently due to tau pathology, and clinicians should be alert to the potential development of progressive supranuclear palsy or corticobasal syndrome.^2^

## Supplementary Material

Teaching Slides
