# Supplementary material for: Teaching NeuroImages: Nonfluent variant primary progressive aphasia: A distinctive clinico-anatomical syndrome
Source: Neurology. 2016 Dec 6;87(23):e283. doi: 10.1212/WNL.0000000000003408 (PMC5177672; doi:10.1212/WNL.0000000000003408)
Supplement: Teaching Slides [file supp_87_23_e283__index.html]

Teaching Slides 

# Teaching Neuro*Images*: Nonfluent variant primary progressive aphasia

## Teaching Slides

**Neurology® data supplements are not copyedited before publication. Published editorials and translations have been copyedited.  
 © 2016 American Academy of Neurology.  
  
 Files in this Data Supplement:**

- Data Supplement - PowerPoint Presentation
