# Supplementary material for: Teaching NeuroImages: Nonfluent variant primary progressive aphasia: A distinctive clinico-anatomical syndrome
Source: Neurology. 2016 Dec 6;87(23):e283. doi: 10.1212/WNL.0000000000003408 (PMC5177672; doi:10.1212/WNL.0000000000003408)
Supplement: Teaching Slides [file supp_WNL.0000000000003408_Teaching_Slides.pptx]

## Slide 1
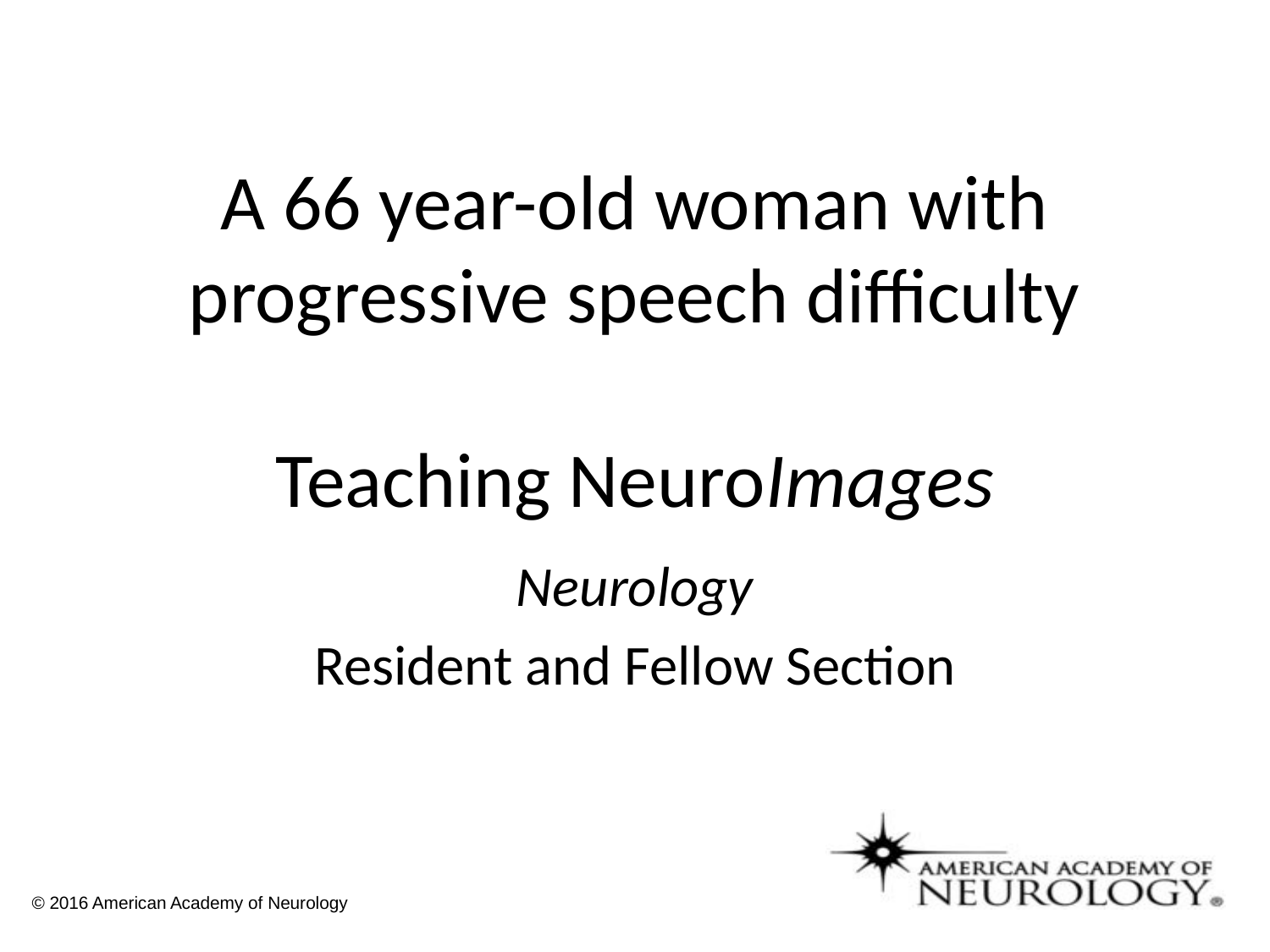

# A 66 year-old woman with progressive speech difficulty
Teaching NeuroImages
Neurology
Resident and Fellow Section
© 2016 American Academy of Neurology

## Slide 2
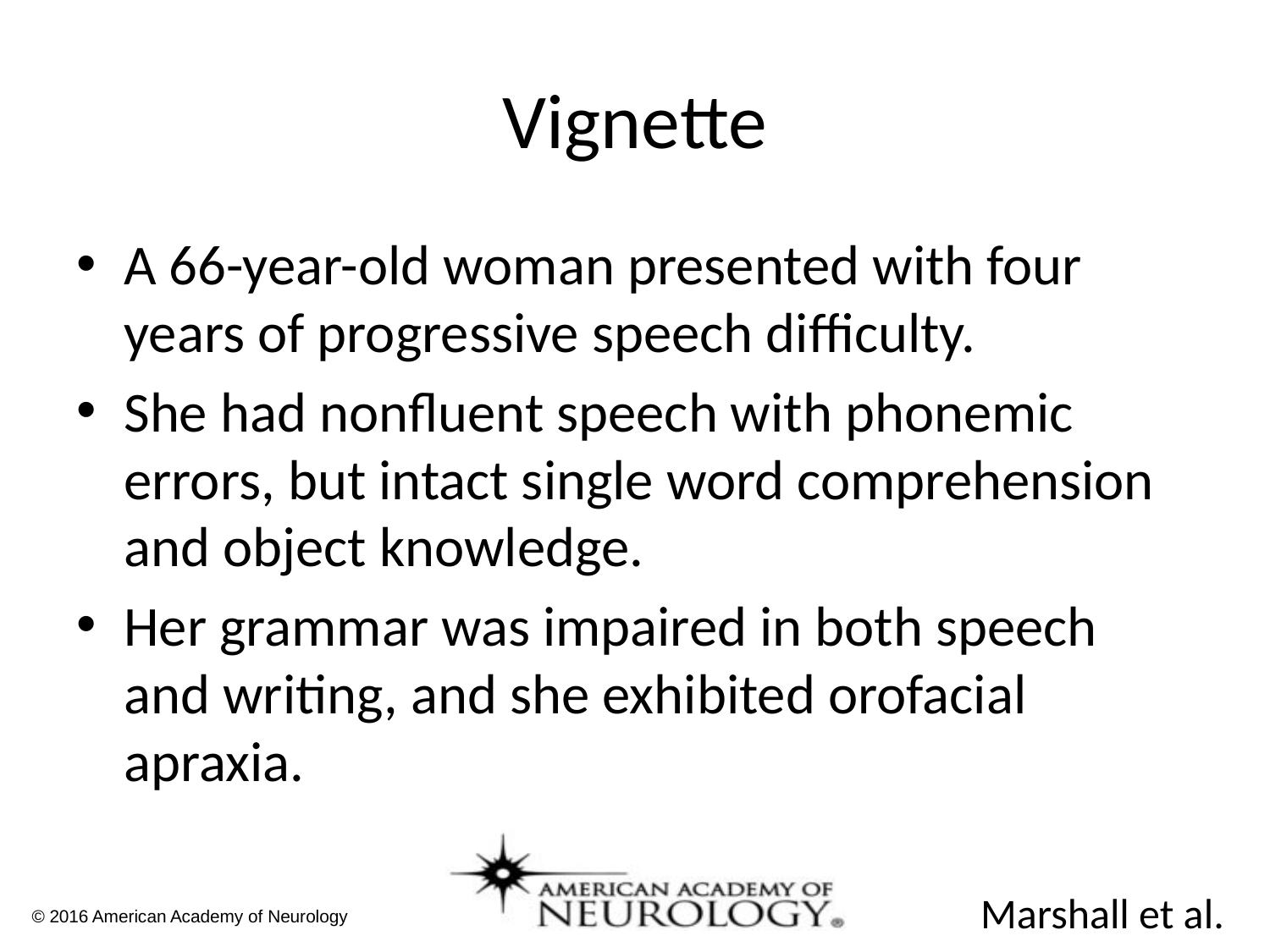

# Vignette
A 66-year-old woman presented with four years of progressive speech difficulty.
She had nonfluent speech with phonemic errors, but intact single word comprehension and object knowledge.
Her grammar was impaired in both speech and writing, and she exhibited orofacial apraxia.
Marshall et al.
© 2016 American Academy of Neurology

## Slide 3
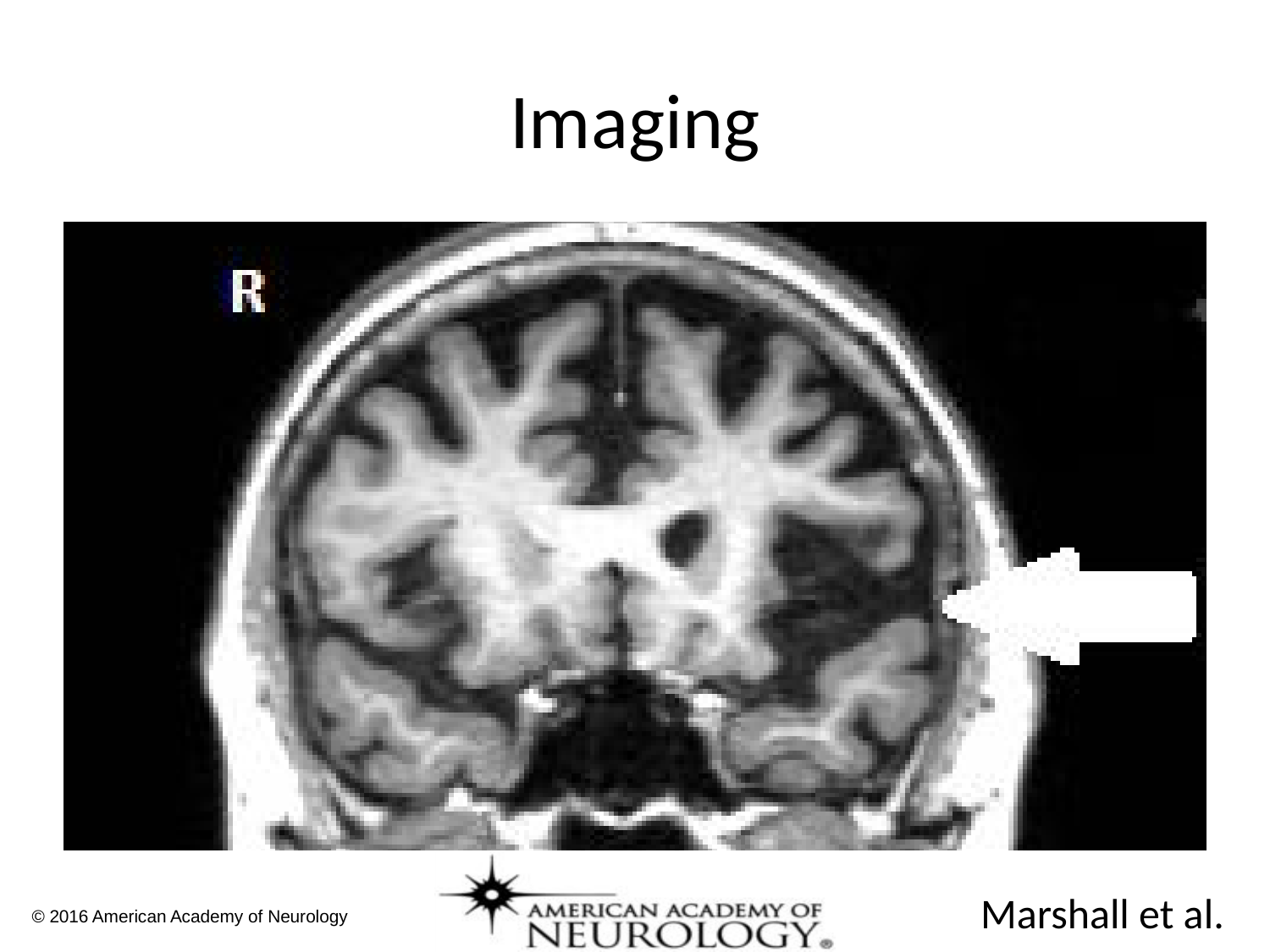

# Imaging
Marshall et al.
© 2016 American Academy of Neurology

## Slide 4
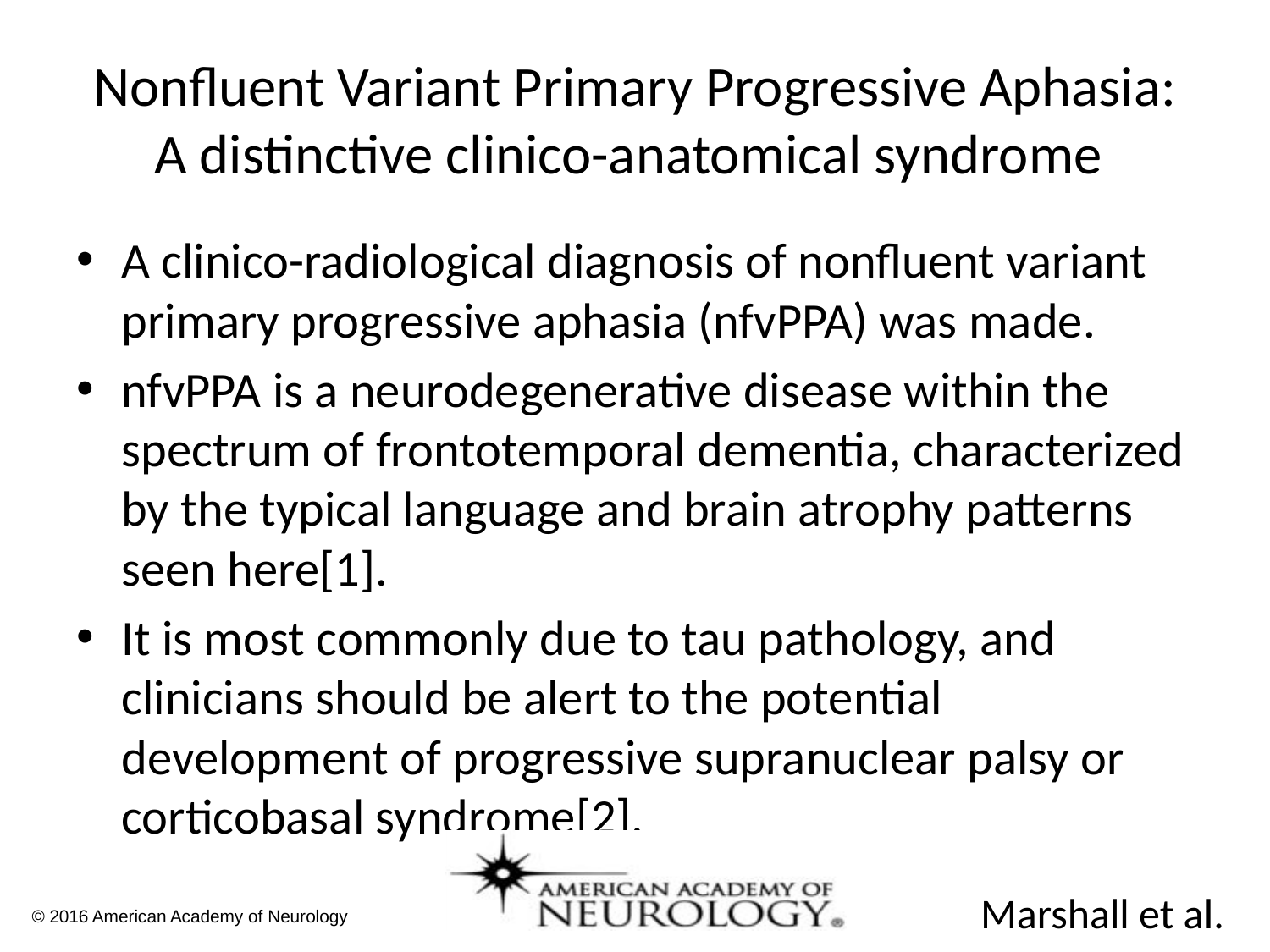

# Nonfluent Variant Primary Progressive Aphasia: A distinctive clinico-anatomical syndrome
A clinico-radiological diagnosis of nonfluent variant primary progressive aphasia (nfvPPA) was made.
nfvPPA is a neurodegenerative disease within the spectrum of frontotemporal dementia, characterized by the typical language and brain atrophy patterns seen here[1].
It is most commonly due to tau pathology, and clinicians should be alert to the potential development of progressive supranuclear palsy or corticobasal syndrome[2].
Marshall et al.
© 2016 American Academy of Neurology
